# Fitness and Dietary Supplements: A Cross-Sectional Study on Food Practices and Nutrivigilance

**DOI:** 10.3390/nu16223928

**Published:** 2024-11-18

**Authors:** Aziz Galman, Mourad Chikhaoui, Mohamed Bouhrim, Bruno Eto, Abdelaaty A. Shahat, Rashed N. Herqash, Rachid Lotfi, Hind Belamgharia, Daoud Daoudi, Morad Kaddouri, Charaf Dlimi, Hassan Alahyane, Habiba Liba, Mohamed Reda Kachmar, Khalid Boutoial

**Affiliations:** 1 Laboratory of the Engineering and Applied Technologies, Higher School of Technology, Sultan Moulay Slimane University, Beni Mellal 23000, Morocco; morad.kaddouri@usms.ma (M.K.); charaf.dlimi@usms.ma (C.D.); khalid.boutoial@usms.ma (K.B.); 2 High Institute of Nursing Professions and Health Techniques, Beni Mellal 23000, Morocco; chimoura7@gmail.com (M.C.); rchdltfi@gmail.com (R.L.); belamghariahind@gmail.com (H.B.); alahyanerh@gmail.com (H.A.); kachmar.reda@gmail.com (M.R.K.); 3 Laboratory of Ecology and Environment, Faculty of Sciences Ben M’Sik, Hassan II University, Casablanca 20023, Morocco; 4 Biological Engineering Laboratory, Faculty of Sciences and Techniques, Sultan Moulay Slimane University, Beni Mellal 23000, Morocco; m.bouhrim@usms.ma; 5 Laboratories TBC, Laboratory of Pharmacology, Pharmacokinetics, and Clinical Pharmacy, Faculty of Pharmaceutical and Biological Sciences, B.P. 83, 59000 Lille, France; 6 Department of Pharmacognosy, College of Pharmacy, King Saudi University, Riyadh 11451, Saudi Arabia; ashahat@ksu.edu.sa (A.A.S.); rherqash@ksu.edu.sa (R.N.H.); 7 Physiology and Pathophysiology Team, Center for Human Pathologies Genomics, Faculty of Sciences, Mohammed V University, Rabat 10050, Morocco; daoud.daoudi@um5r.ac.ma; 8 Higher Institute of Nursing Professions and Health Techniques of Marrakesh, Marrakech 40000, Morocco; habibalibasante@gmail.com; 9 Valorization of Medicinal and Aromatic Plants and Environment Team, Faculty of Sciences, Moulay Ismail University, Meknes 50000, Morocco

**Keywords:** dietary supplement, fitness, adverse effects, nutrivigilance, food practices

## Abstract

**Background/Objectives:** The use of dietary supplements (DSs) has become common among fitness enthusiasts, aiming to enhance performance, recovery, and overall well-being. **Methods**: A cross-sectional study was conducted in the city of Beni Mellal from April to July 2024, assessed dietary practices, motivations for supplement use, and associated adverse effects among 420 survey participants. **Results:** The majority of dietary supplement users were aged 25–64 and had higher education levels. Colopathy (67.38%) was the most common eating disorder, followed by digestive (59.46%), inflammatory, and rheumatic diseases (53.50%). Dietary supplementation prevalence was 88.1%, with proteins (60.81%), medicinal plants (45.13%), and vitamins (42.70%) being the most consumed. Key motivations included supporting muscle, bone, and joint strength (musculoskeletal) (83.78%) and enhancing heart and lung function for cardiorespiratory health (82.43%). However, 28% of protein users experienced adverse effects, such as myalgia, gastralgia, palpitations, and insomnia. Multivariate linear regression indicated a significant negative association of creatine with effectiveness (β = −0.485, *p* = 0.001). **Conclusions**: Overall, while the benefits of dietary and sports practices are evident, the adverse effects associated with protein supplements highlight the necessity for enhanced nutrivigilance and nutritional education to ensure safe supplements.

## 1. Introduction

Dietary supplement (DS) consumption in fitness centers has increased significantly over the past few decades [1]. In 2023, the global sports nutrition supplement market was valued at approximately USD 15.6 billion, with an expected annual growth rate of 4.8% through 2028 [2]. Commonly used supplements include protein powders, creatine, bodybuilding branched-chain amino acids (BCAAs) such as leucine, isoleucine, and valine [3], vitamins and minerals, and omega-3 [4]. These supplements are often consumed to enhance athletic performance, speed up recovery, and support muscle growth [5]. However, their use may pose health risks, as some studies have revealed the presence of illegal doping substances or contaminants in certain products, potentially causing serious adverse effects (AEs) [6]. For example, the French National Agency for Food, Environmental, and Occupational Health and Safety (ANSES) has reported cases of fatalities and serious health complications linked to the use of 2,4-dinitrophenol as a weight loss supplement in sports [7]. The most common adverse effects are cardiovascular in nature, including tachycardia, palpitations, and even cardiac arrest. Several studies have highlighted the link between DS consumption and adverse effects, particularly among athletes [8,9]. Approximately 20% of these supplements contain undisclosed pharmacologically active compounds [10], increasing the risk of negative health outcomes. Commonly reported side effects from over-consumption include gastrointestinal issues such as nausea, abdominal cramps, and diarrhea [4]. Additionally, some DSs may be contaminated with banned substances, like anabolic steroids, which can cause hormonal imbalances and cardiovascular complications [11]. Excessive intake of stimulant-containing products, such as caffeine, BCAAs, and creatine, has been linked to an elevated risk of myopathy, a condition that can range from mild, asymptomatic increases in creatine kinase (CK) and muscle weakness, aches, and fatigue to severe cases, including rhabdomyolysis, a serious condition that may lead to kidney damage (Williams and Thorpe 2014; Orellana-Valdez et al. 2023) [12,13]. Moreover, regular use of performance-enhancing supplements can result in psychological dependence among athletes [14].

In Morocco, the dietary supplements market is estimated at approximately USD 12 million, with an annual growth rate of 25% since 2015 according to the Minister of Industry and Commerce [15,16]. However, few studies have investigated dietary supplement use among athletes, leaving limited data on supplement consumption in fitness centers. Kartouti and Khalfaoui [15] found that about 56.29% of Moroccan fitness enthusiasts use dietary supplements to enhance athletic performance. The most commonly used products include protein powders, BCAAs, and multivitamins [16]. However, concerns persist about the quality and safety of these supplements due to unreported adverse reactions, exacerbated by the absence of a national reporting system.

To enhance the quality control of dietary supplements in the Moroccan market, a recent regulatory convention was signed by the Ministry of Health and Social Protection (MHSP) and the Ministry of agriculture, Sea Fishing, Rural Development, Water and Forests (MASFRDWF). This convention establishes strict standards, provisions, and guidelines for marketing dietary supplements, whether locally or imported [17]. However, regulations remain relatively lenient, permitting supplements to be sold in public venues such as gyms without requiring expertise from retailers or stringent oversight of product origins. This lack of control increases the risk of counterfeit products, raising concerns about the safety and quality of dietary supplements available on the national market [18].

The safety of DSs on human health has drawn the attention of various international organizations, including ANSES, which first introduced the concept of “Nutrivigilance”. This refers to a monitoring system that collects and analyzes information on adverse effects associated with DSs [19]. Its primary goal is to safeguard consumer health by swiftly identifying any harmful effects of these products [20]. Similarly, the World Health Organization (WHO) works to enhance food safety by providing information on potential risks related to specific ingredients or formulations [21]. The data collected through these systems help public health authorities make informed regulatory decisions while aiding investigations into the effects of DSs. Such vigilance frameworks are crucial for protecting population health and ensuring the safety and quality of food products.

In Morocco, no studies have been conducted to evaluate the nutrivigilance of dietary supplements, whether among athletes or the general population. However, establishing a nutrivigilance system is essential for monitoring dietary supplement use, focusing on quality, nutritional education, and adverse effect surveillance that participate to enhance consumer safety, as seen in countries that have implemented such systems, including in France, Italy, and the USA [20,22]. Therefore, the current study aims to thoroughly investigate the consumption practices of dietary supplements among fitness enthusiasts in Beni Mellal City. It will focus on analyzing their dietary habits, and nutritional behaviors, and identifying any adverse effects associated with these products. The originality and significance of this research lie in its pioneering exploration of this topic, providing essential information that can improve dietary practices among fitness enthusiasts and address gaps in knowledge in this critical area.

## 2. Materials and Methods

### 2.1. Study Design and Area

A cross-sectional survey was conducted from April to July 2024 across five fitness clubs in Beni Mellal City to explore the practices, motivations, and adverse effects behind supplement consumption among regular fitness enthusiasts. This city was chosen due to its extensive availability of fitness centers, pharmacies, and parapharmacies, as well as its numerous large commercial areas.

### 2.2. Study Population, Sample Size, and Data Collection

Of the more than 2000 fitness enthusiasts initially invited to participate, only 420 gave consent and were accepted for the survey. The process began with the validation of the final questionnaire, assessing its organization, the clarity of scientific terms related to dietary supplements and fitness activities, its appropriateness, and completeness. The questionnaire was then tested on a sample group of fitness enthusiasts, and modifications were made based on the feedback received during this phase. The survey was created in French, and both the pilot and final studies were conducted through interviews in French. Data were gathered through face-to-face interviews, where each participant was informed of the study’s objectives, content, and expected outcomes, and each participant provided their consent. The data from the pilot study were not used in the final analysis. The study included individuals over the age of 15 who regularly engaged in fitness activities and were familiar with dietary supplementation.

### 2.3. Survey

The survey was created based on a comprehensive review of the existing literature. It was divided into two sections:

The first section focused on specific demographic characteristics, gathering information on age, gender, educational level, preferred sport, duration of sports practice, dietary habits, eating disorders (EDs), medical history, Body Mass Index (BMI), and the use of dietary supplements.

In the second section, participants who reported using dietary supplements were asked specific questions regarding the types of supplements they used, their compositions, duration of use, reasons for consumption in both sports and non-sports contexts, as well as their perceived effectiveness and any associated adverse effects. The questionnaires were distributed across five large fitness studios from April to July over a span of four months.

### 2.4. Statistical Analysis

Statistical analysis was performed using SPSS for Windows, version 25.0. Descriptive data were presented as the mean for interval data and as percentages and proportions for categorical data. Multivariate linear regression analysis was conducted to assess the impact of several predictors (types of supplements, duration of consumption, duration of fitness activity, diet, eating disorders, pathological status, etc.) on the efficacy of dietary supplements in relation to sports performance and well-being among fitness enthusiasts. Both unstandardized coefficients (B) and standardized coefficients (Beta) were included to show the direction and strength of each predictor’s effect. Also, 95% confidence intervals (CIs) were reported for the regression results, with statistical significance set at *p* < 0.05 for all analyses.

To evaluate the construct validity and reliability of the questionnaire, we conducted a factor analysis on our sample, which identified five primary factors influencing dietary supplementation practices. The Kaiser–Meyer–Olkin (KMO) test was used to assess the adequacy of the data for the factor analysis, yielding a KMO value of 0.724, which suggests a satisfactory level of correlation among the items in the questionnaire. Additionally, Bartlett’s test of sphericity indicated a chi-square value of 5541.510 with 276 degrees of freedom (*p* < 0.001), further confirming the significance of the correlations between the variables. The analysis revealed that the eigenvalues of the extracted factors indicated that the first factor explained 21.58% of the total variance, while the second factor accounted for 15.32%, bringing the cumulative variance explained by these two factors to 36.91%. Furthermore, the overall Cronbach’s alpha for all items was measured at 0.837, demonstrating good internal consistency within the questionnaire. These findings provide robust evidence for the questionnaire’s construct validity and reliability in assessing dietary supplementation practices.

## 3. Results

### 3.1. Sociodemographic Characteristics

Out of 420 regular fitness practitioners recruited from five fitness centers in Beni Mellal City, the largest group fell within the 25–64 age range, accounting for 216 individuals (51.43%), followed by the 15–24 age group with 199 individuals (47.38%), and those over 65 years accounted for 5 individuals (1.19%). The majority of the participants were male with 360 (85.71%) and 60 females (14.29%), resulting in a sex ratio of 6. In terms of education, 255 participants (60.71%) were pursuing higher education, 105 (25%) were at the secondary level, 59 (14.05%) were in middle school, and 1 participant (0.24%) had only completed primary education (Table 1). The majority of participants engaged in stretching exercises, with 409 participants (97.38%) practicing these routines. This was followed by 393 participants (93.57%) who performed aerobic exercises. Nearly half of the participants, 235 participants (55.95%), also incorporated weight training into their fitness regimen. In terms of fitness experience, the majority were advanced practitioners with over three years of experience (*n* = 285, 67.86%), followed by intermediate practitioners (*n* = 85, 20.24%), and beginners with less than one year of experience (*n* = 50, 11.90%).

### 3.2. Pathological Status of the Studied Population

The eating disorders and health conditions of the studied population are summarized in Table 2. Colopathy was the most common eating disorder, affecting 283 participants (67.38%), followed by anorexia, which was observed in 43 participants (10.24%). Regarding the pathology profile, the most common diseases were digestive diseases, affecting 242 participants (57.62%), followed by inflammatory and rheumatic diseases in 224 participants (53.33%), and neuropathies in 142 participants (33.81%). Other reported conditions included urogenital pathologies (*n* = 60, 14.29%), cardiovascular diseases (*n* = 32, 07.62%), and respiratory and allergic diseases affecting 33 participants (07.86%) (Table 2).

### 3.3. Dietary Profile and Consumption of Dietary Supplements Among Participants

Among the 420 participants, 370 practitioners reported using DSs. The majority were omnivores (*n* = 305, 82.43%), followed by vegetarians (*n* = 40, 10.81%), and vegans (*n* = 25, 6.76%). The most commonly used DSs included proteins and amino acids (*n* = 225, 60.81%), medicinal plants (*n* = 167, 45.13%), and vitamins (*n* = 158, 42.70%). Other supplements used included minerals (*n* = 137, 37.02%), fatty acids (*n* = 110, 29.72%), and bee products (*n* = 103, 27.83%). Most participants consumed DSs for over three months (*n* = 272, 73.51%) (Table 3).

### 3.4. Motivation for Consuming Dietary Supplements

The motivations and daily reasons for consuming dietary supplements among the 370 users are gathered in Table 4. A total of 310 participants (83.78%) reported using dietary supplements to strengthen the musculoskeletal system, followed by cardiorespiratory health (*n* = 305, 82.43%), bodybuilding (*n* = 170, 45.94%), and weight loss (*n* = 54, 14.59%). Regarding daily reasons for DS consumption, 350 (94.59%) consumers indicated that their primary goal was to improve their physical and biological health, while 316 (85.41%) aimed to enhance sports performance, and 232 (62.70%) sought to maintain body esthetics.

### 3.5. Dietary Practices and Supplementation Effectiveness

The results of the multiple linear regression, shown in Table 5, examine the relationship between DS effectiveness and various predictors. The data reveal that certain supplements and usage duration are significant predictors of perceived supplementation effectiveness. Vitamin supplements (B = 0.450, β = 0.208, *p* = 0.001), fatty supplements (B = 0.205, β = 0.086, *p* = 0.014), dietary medicinal plants (B = 0.326, β = 0.152, *p* < 0.001), and particularly the duration of dietary supplementation (B = 0.425, β = 0.646, *p* < 0.001) all show positive associations with effectiveness, suggesting that these supplements and longer usage enhance the perceived benefits. Conversely, protein supplements exhibit a significant negative association (B = −0.562, β = −0.267, *p* < 0.001), possibly due to overuse or a mismatch with user goals. Other factors, such as BMI, diet, duration of sporting activity, and certain supplement types (e.g., mineral, sea, and non-revivable yeast), did not show significant effects, indicating minimal impact on perceived effectiveness. Confidence intervals for significant predictors reinforce these findings, suggesting that targeted supplements and consistent use are key to perceived effectiveness.

### 3.6. Nutrivigilance and Protein Supplementation

The results of the multiple linear regression, as shown in Table 6, underscore the relationship between protein supplementation and the well-being of fitness enthusiasts. While significant effects were identified for two types of protein supplements, no notable effects were found for others. Specifically, keratin supplementation showed a significant positive association with effectiveness (B = 0.674, β = 0.133, *p* = 0.019), suggesting that its consumption improves supplementation outcomes. In contrast, creatine exhibited a highly significant negative association with effectiveness (B= −0.485, β = −0.193, *p* = 0.001). This finding aligns with participants’ reports of adverse effects from protein supplement use, including gastralgia, dehydration, fatigue, myalgia, insomnia, and, in some cases, palpitations or side stitches during exercise; these symptoms did not exist before supplement use. These results emphasize the importance of nutrivigilance, which aims to enhance consumer safety by identifying and monitoring potential adverse effects associated with the consumption of certain food products, such as dietary supplements.

## 4. Discussion

The present study is a cross-sectional survey conducted in five fitness centers in Beni Mellal City to describe the sporting and dietary practices of fitness enthusiasts, also examining the impact of dietary supplementation on their effectiveness (performance and well-being), including any potential adverse effects. Given that sporting performance, as scientifically defined, encompasses physical, psychological, and biological dimensions that interact with the concept of well-being, it is essential to analyze the results of this study by linking these two notions in the context of supplement efficacy among fitness enthusiasts. Recent psychometric research supports this approach; for instance, the Italian version of the tempest self-regulation questionnaire for Eating and the Behavioral Regulation in Exercise Questionnaire (BREQ-3) demonstrated reliability, validity, and measurement invariance, affirming their utility in assessing self-regulation in eating and exercise behaviors within fitness contexts, underscoring the importance of self-regulation in dietary and exercise habits, which directly impact the effectiveness of supplements on physical performance and well-being [23,24]. The majority of participants in this study were male, which aligns with the findings from numerous studies showing a male predominance in dietary supplement consumption, particularly within sports and fitness contexts [4,25]. This indicates that males are more likely to use supplements aimed at enhancing physical performance, increasing muscle mass, and supporting post-exercise recovery. Additionally, participants with higher levels of education were more likely to consume dietary supplements. In fact, recent studies have consistently demonstrated a strong correlation between higher educational attainment and increased supplement use [26,27]. Individuals with a university degree or higher are more likely to take supplements compared to those with lower educational levels. This trend is often attributed to greater health awareness, a higher degree of health literacy, and the financial means to access a wider range of supplements. Our findings indicate that colopathy and anorexia were the most common eating disorders among participants, with digestive, inflammatory, rheumatic, and neurological diseases being the most prevalent health conditions. These results are consistent with other studies. For instance, Keel [28] confirmed the widespread occurrence of functional digestive disorders like colopathy globally. Similarly, Kronbi et al. [29] highlighted the high prevalence of inflammatory and rheumatic diseases in Morocco, particularly among patients using dietary supplements and alternative medicine. Furthermore, the WHO [30] documented frequent neurological disorders in all regions of the world. In terms of diet, omnivorous consumers tend to report greater satisfaction with dietary supplements. Similarly, Lentjes [31] indicated that individuals with organized eating habits are more receptive to the effects of supplements. Conversely, Pohl et al. [32] suggest that diet type (vegan, vegetarian, omnivorous) does not always significantly influence satisfaction with dietary supplements.

These findings indicate that the most commonly consumed products are those rich in protein and amino acids, medicinal plants, and vitamins. This trend aligns with global patterns observed in recent years, especially among athletes and health-conscious individuals looking to improve their physical performance [33,34,35]. Frequently consumed supplements include proteins, amino acids, medicinal plants, and vitamins, each serving specific roles in supporting health and fitness objectives. Kreider et al. [5] noted that protein supplementation, especially with whey and casein, is popular for enhancing muscle mass and improving recovery. Amino acids, particularly BCAAs, are favored for their roles in reducing muscle fatigue and promoting protein synthesis during and after exercise [5]. Medicinal plants like ginseng, echinacea, and green tea have also gained popularity for their potential health benefits. Riaz et al. [36] found that ginseng is commonly used for its energy-boosting effects, while echinacea is often taken to enhance immune function. Vitamins remain among the most widely used dietary supplements globally, with multivitamins being particularly popular for preventing nutrient deficiencies and promoting overall well-being. Bailey et al. 2011 [37] emphasized that multivitamin use is common among individuals aiming to maintain optimal health and prevent chronic diseases. The participants in the current study reported that their primary goals for using dietary supplements (DSs) included strengthening the musculoskeletal system, improving cardiorespiratory health, bodybuilding, and weight loss. This finding is consistent with global trends that reveal similar motivations. For example, Knapik et al. [25] and Rani [38] discovered that athletes often utilize DSs to enhance physical performance, particularly for muscle growth and recovery. Garthe and Maughan [33] similarly reported that athletes and fitness enthusiasts worldwide use supplements to improve musculoskeletal systems and cardiorespiratory health. Moreover, Bailey et al. [26] highlighted that weight loss and general health maintenance are significant drivers of DSs use among the general population. Regarding the daily motivations for DS consumption, participants noted that their primary aim was to enhance their physical and biological health while improving sports performance. This finding aligns with global trends, as demonstrated by Ammar et al. [39], who showed that many adults prioritize supplements to support recovery and boost performance. In addition, Parry et al. [40] revealed that health-conscious individuals often turn to DSs to enhance overall wellness, reflecting a widespread belief in their efficacy. Abreu et al. [41] also noted that both recreational and competitive athletes commonly use DSs for health improvements, suggesting that these motivations span diverse demographic groups. Furthermore, Peeling et al. [42] emphasized the role of nutritional supplementation in achieving fitness goals, reinforcing the idea that health and performance enhancement drives DS consumption. Our findings support these observations, demonstrating significant associations between the effectiveness of specific dietary supplements, particularly vitamins, fatty supplements, and medicinal plants. This underscores the essential role that these supplements play in enhancing overall health and efficacy. For instance, vitamin supplements are widely recognized for their ability to address nutrient deficiencies and improve immune function [43,44]. Fatty supplements, such as omega-3 fatty acids, have been linked to benefits for cardiovascular and cognitive health [45,46]. Furthermore, medicinal plants like ginseng and turmeric are celebrated for their anti-inflammatory and therapeutic properties, which contribute to improved well-being and recovery [47,48].

Despite the reported positive and significant impacts of dietary supplements, such as enhanced sports performance, some consumers experience unmet expectations, particularly concerning adverse effects in long-term users. This issue is notably prevalent among those who favor protein supplements, especially creatine. These findings highlight the need to strengthen nutrivigilance practices and improve consumer education to better align expectations with the actual effects of dietary supplements. The participants in our study reported side effects from supplement consumption, including gastralgia, dehydration, and fatigue, consistent with other studies. For example, Fernández-Landa et al. [49] observed slight negative effects of supplements on endurance in trained populations, while Poortmans and Francaux [50] noted that excessive intake of amino-group substances like creatine can strain the liver and kidneys. A case study described a 27-year-old male who developed jaundice after consuming creatine for eight to nine months and whey protein four weeks prior to symptom onset [51]. Creatine intake increases serum creatinine levels as it spontaneously converts to creatinine, potentially leading to false diagnoses of kidney damage when assessed solely by blood tests [52]. Additionally, the literature controversially links creatine consumption with muscle cramps, which may result from increased intracellular osmotic load due to creatine and worsened by dehydration during physical activity [53]. The exact mechanisms underlying these adverse effects remain unknown. In contrast, Antonio et al. [54] indicate that creatine does not significantly impact hydration status or kidney health in healthy individuals. In contrast to the adverse effects observed with creatine among our participants, keratin supplementation showed a significant positive association with effectiveness, suggesting that its consumption enhances supplementation outcomes. This is similar to the results of McLeay et al.’s study [55], who reported that athletes may benefit from maintaining or increasing lean body mass by consuming keratin supplements. They also noted that a keratin-based protein supplement is comparable to casein in increasing lean body mass among trained male cyclists and was both safe and well tolerated. The findings of this study indicate a non-significant correlation for BCAAs. However, BCAAs have previously been shown to be beneficial for muscle recovery and reduce fatigue [56]. Contrarily, Holeček [57] challenged this view, demonstrating that BCAAs can lead to adverse effects such as gastrointestinal disorders, metabolic imbalances, and even cardiovascular disease. According to surveys by the Office of Dietary Supplements (ODSs), adverse effects like nausea and stomach upset are most commonly reported in individuals who consume BCAAs in excess [58]. These observations underscore the adverse effects of overuse and highlight the need for a balanced and moderate intake.

The adverse effects reported by the ANSES nutrivigilance system were predominantly cardiovascular, with less frequent psychiatric, hepatic, nephrological, neurological, dermatological, and gastroenterological issues. These findings align with our analysis of intolerable effects linked to protein supplements. Vigilance regarding these products focuses on monitoring interactions during the simultaneous intake of multiple proteins, such as avoiding the prohibited combination of proteins with substances like caffeine, p-synephrine, or amphetamine-type stimulants [59]. Concerns also arise from combinations such as whey, amino acids, and creatine, as well as the use of counterfeit substances like 2,4-DNP [60], sibutramine and anabolic androgenic steroids [61], and dimethylamylamine (DMAA) [62]. The ANSES has confirmed through academic studies that taking creatine alongside BCAAs negatively impacts both sports performance and well-being, as creatine diminishes the effectiveness of valine. Monitoring regulatory compliance in product marketing is essential to ensure consumer safety.

To reduce adverse effects from dietary supplement consumption in Morocco, as highlighted in our study, it is crucial to establish a nutrivigilance system to safeguard consumers from risks related to food and supplement use. Similar systems have proven to be highly effective worldwide. France’s ANSES, one of Europe’s strongest nutrivigilance systems since 2009, uses standardized protocols for reporting and analyzing adverse effects, improving consumer safety with timely advisories [20,63,64]. Scandinavian countries like Sweden and Denmark have adopted rigorous standards, although nutrivigilance practices are more stringent for traditional foods and medicines than for dietary supplements. Denmark’s approach, for instance, involves the Danish Veterinary and Food Administration (DVFA), which oversees food safety and supplements [20]. Scandinavian systems benefit from high consumer awareness and stringent health regulations, although they too face limitations in cross-national data sharing. In Spain, the Spanish Agency for Food Safety and Nutrition (AESAN) enables adverse reaction reporting and collaborates with healthcare professionals to evaluate potential hazards [65]. In the United States, the Food and Drug Administration (FDA) enables healthcare providers, consumers, and manufacturers to report adverse events related to supplements, facilitating rapid risk detection, though it faces challenges from underreporting and minimal pre-market testing [66]. To enhance nutrivigilance in Morocco, it is essential to expand on the proposed strategies by incorporating effective policies from other regions. Establishing a national nutrivigilance system similar to those in the aforementioned countries would enable the monitoring of adverse effects from dietary supplements and ensure a swift response to health risks. Strengthening collaboration among stakeholders would enhance data collection and increase accountability among manufacturers. Furthermore, public awareness campaigns regarding the safe use of food products, akin to those implemented in France, Scandinavian countries, and others, could inform consumers about potential risks. Implementing standardized reporting procedures, as recommended by the WHO, would streamline data collection and analysis of adverse events. Additionally, leveraging digital technologies for reporting and providing ongoing training for healthcare professionals to recognize adverse effects are crucial steps to consider. By drawing on these international examples, Morocco could significantly bolster its nutrivigilance framework, thereby safeguarding consumer health and building trust in the available food products.

Despite the strengths of this study, several limitations should be acknowledged. While data were collected from a large sample of young people and adults over 15 years of age in Beni Mellal City, future research across different regions of Morocco would be valuable to generalize the findings. Additionally, as data are self-reported, there is potential for recall bias (under- or overestimation) and social desirability bias (due to fears or misperceptions). The cross-sectional design limits the ability to establish causal relationships, and the lack of biological measures restricts the objective assessment of supplement effects on health. Future research could enhance our understanding of dietary supplement effectiveness by experimentally evaluating the quality of marketed supplements and employing representative samples with longitudinal methodologies to strengthen and expand upon these conclusions.

## 5. Conclusions

Further research is critical to advancing our understanding of the adverse effects (AEs) of dietary supplements, particularly protein-based ones, among fitness enthusiasts. This study contributes to the growing need for vigilant monitoring in supplement use within sports activities, emphasizing the gap between consumer expectations and the actual effects perceived. Given the high prevalence of dietary supplement use and the associated adverse effects, there is a pressing need for a national nutrivigilance system to regulate and monitor the safety of sports supplements, including proteins and vitamins, to mitigate health risks. Our findings also underscore the importance of educational programs focused on safe supplement practices. Public health initiatives could promote targeted awareness campaigns in fitness centers and educational institutions, equipping individuals with knowledge on safe consumption practices and helping to prevent common health issues, such as digestive, cardiovascular, and renal problems. By integrating regulatory measures with public health education, policymakers can better support the well-being of Morocco’s active population, fostering safer fitness practices in line with the national health objectives.

## Figures and Tables

**Table 1:** Sociodemographic characteristics, types of fitness activities, and fitness experience of the studied population.

Variables	Absolutely Frequency (*n*)	Relative Frequency (%)
**Age groups (years)**		
15–24	199	47.38
25–64	216	51.43
≥65	5	1.19
**Gender**		
Male	360	85.71
Female	60	14.29
**Level of education**		
Primary	1	0.24
Secondary middle school	59	14.05
Secondary high school	105	25.00
Higher education	255	60.71
**Fitness type**		
Stretching exercises	409	97.38
Aerobic exercises	393	93.57
Weight training	235	55.95
**Fitness experience (years)**		
>1	50	11.90
1–3	85	20.24
<3	285	67.86

**Table 2:** Eating disorders and pathological status of the studied population.

Variables	Absolutely Frequency (*n*)	Relative Frequency (%)
**Eating disorder**		
Colopathy	283	67.38
Anorexia	43	10.24
Intestinal malabsorption	29	6.90
Bulimia	17	4.05
**Pathology profile**		
Digestive diseases	242	57.62
Inflammatory and rheumatic diseases	224	53.33
Neuropathy	142	33.81
Urogenital diseases	60	14.29
Respiratory and allergic diseases	33	7.86
Cardiac diseases	32	7.62
Hemopathy	28	6.67
Dermatologic diseases	22	5.24
Autoimmune diseases	13	3.10

**Table 3:** Dietary habits, types of dietary supplements, and duration of consumption among the studied population.

Variables	Absolutely Frequency (*n*)	Relative Frequency (%)
**Diet**		
Omnivorous	305	82.43
Vegetarian	40	10.81
Vegan	25	6.76
**Dietary supplement type**		
Proteins/amino acids	225	60.81
Vitamins	158	42.70
Medicinal plants	167	45.13
Minerals	137	37.02
Fatty acids	110	29.72
Bee products	103	27.83
Probiotics and yeasts	84	22.70
Sea products	58	15.67
**Consumption period of dietary supplement (months)**		
≤3	08	2.16
4–5	90	24.33
≥6	272	73.51

**Table 4:** Motivation and daily reasons for consuming DSs among the studied population.

Variables	Absolutely Frequency (*n*)	Relative Frequency (%)
**Sportive motivation**		
Strengthen the musculoskeletal (muscles, bones, and joints)	310	83.78
Cardiorespiratory health (improve heart and lung function)	305	82.43
Bodybuilding	170	45.94
Lose weight	54	14.59
**Daily reasons for consuming dietary supplements**		
Physical and biological health	350	94.59
Sportive performance	316	85.41
Beauty and cosmetics	232	62.70
Reproductive and sexual health	64	17.30

**Table 5:** Dietary supplements effectiveness and other predictors (food and sports practices) were assessed by multiple linear regression.

Independent Variables	Unstandardized Coefficients, B (CI *)	Standardized Coefficients, β	*p*
Body Mass Index (kg/m^2^)	0.275 (−0.999, 1.549)	0.013	0.671
Diet	0.088 (−0.021, 0.196)	0.051	0.113
Duration of sporting activity	0.022 (−0.066, 0.11)	0.015	0.624
Eating disorders	0.014 (−0.146, 0.174)	0.005	0.865
Pathology profile	−0.132 (−0.306, 0.042)	−0.048	0.136
Vitamin supplements	0.450 (0.19, 0.711)	0.208	0.001 **
Mineral supplements	−0.014 (−0.282, 0.253)	−0.006	0.916
Protein supplements	−0.562 (−0.733, −0.391)	−0.267	0.000 **
Fatty supplements	0.205 (0.041, 0.368)	0.086	0.014 **
Dietary medicinal plants	0.326 (0.174, 0.479)	0.152	0.000 **
Beehive supplements	0.138 (−0.019, 0.295)	0.057	0.084
Sea supplements	−0.004 (−0.194, 0.187)	−0.001	0.970
Pre-probiotic supplements	0.178 (−0.011, 0.367)	0.056	0.065
Non-revivable yeast supplements	0.074 (−0.183, 0.332)	0.018	0.571
Duration of DSs	0.425 (0.37, 0.481)	0.646	0.000 **

* CI: 95% confidence interval, **: statistically significant at the 0.05 level.

**Table 6:** Impact of protein supplements on sports well-being assessed using multiple linear regression.

Independent Variables	Unstandardized Coefficients, B (CI *)	Standardized Coefficients, β	*p*
Whey (Lactoserum)	−0.067 (−0.368, 0.233)	−0.029	0.660
Casein	1.276 (−0.78, 3.332)	0.059	0.223
Arginine	0.18 (−0.194, 0.554)	0.05	0.345
Keratin	0.674 (0.11, 1.237)	0.133	0.019 **
Glutamine/L-glutamine	−0.109 (−0.486, 0.268)	−0.031	0.571
Leucine, isoleucine, valine	−0.109 (−0.378, 0.159)	−0.047	0.423
Taurine	0.252 (−0.203, 0.707)	0.059	0.277
Creatine	−0.485 (−0.768, −0.203)	−0.193	0.001 **
Collagen	0.169 (−0.411, 0.749)	0.033	0.568
Methionine	−0.175 (−1.134, 0.783)	−0.018	0.719
Tryptophan	0.142 (−0.188, 0.472)	0.047	0.398

* CI: 95% confidence interval, **: statistically significant at the 0.05 level.

## Data Availability

The original contributions presented in this study are included in the article. Further inquiries can be directed to the corresponding author.
